# Associations Between Blunted Pupillary Constriction and Dilation in Individuals with Schizophrenia: Evidence of a Common Mechanism?

**DOI:** 10.1093/schizbullopen/sgaf017

**Published:** 2025-08-20

**Authors:** Christophe Delay, Jan W Brascamp, Jessica Fattal, Matthew Lehet, Beier Yao, Katharine N Thakkar

**Affiliations:** Department of Psychology, Michigan State University, East Lansing, MI 48825, United States; Department of Psychology, Michigan State University, East Lansing, MI 48825, United States; Department of Psychology, Northwestern University, Evanston, IL 60208, United States; Department of Psychology, Chatham University, Pittsburgh, PA 15232, United States; Schizophrenia and Bipolar Disorder Program, McLean Hospital 115, Belmont, MA 02478, United States; Department of Psychiatry, Harvard Medical School, Boston, MA 02115, United States; Department of Psychology, Michigan State University, East Lansing, MI 48825, United States

**Keywords:** psychophysiology, biomarkers, illness mechanisms, pupillary dynamics

## Abstract

**Background and Hypothesis:**

Unpacking the pathophysiological mechanisms of schizophrenia is necessary for advancing prediction, prevention, and treatment efforts. Mechanisms can be identified using easy-to-use and scalable clinical biomarkers, which reflect illness processes. Pupillometry is one such biomarker. Blunted dilation related to cognitive demand has been interpreted as a metric of diminished effort in schizophrenia, while blunted constriction to light has been interpreted as a metric of altered autonomic balance in schizophrenia. However, these 2 sets of findings may also reflect a common mechanism of schizophrenia. Therefore, this study aimed to explore the association between blunted cognitive dilation and blunted constriction to light to provide a parsimonious mechanism of autonomic and effort disturbances experienced by individuals with schizophrenia.

**Study Design:**

We assessed light-induced constriction and cognitive dilation during a double-step task in individuals with schizophrenia (*n* = 84) and demographically matched healthy controls (HC, *n* = 69), compared these metrics between groups, and computed their correlation within each group.

**Study Results:**

Replicating prior findings, dilation and constriction were blunted in schizophrenia relative to HC. Blunted constriction and dilation were positively correlated in schizophrenia but not HC (although the 2 correlations did not differ significantly).

**Conclusions:**

Findings provide, for the first time, preliminary support of a common mechanism linking blunted pupil constriction to light and dilation to cognitive demands in schizophrenia. We propose that individuals with schizophrenia may exhibit impaired top-down modulation of autonomic control. Future studies are needed to validate this proposed mechanism.

## Introduction

Biomarkers are essential for improving diagnostic accuracy, prediction, prevention, and treatment of schizophrenia. For a biomarker to be clinically meaningful, however, certain criteria must be met.^1^ A biomarker must reflect central pathophysiological processes (rather than, eg, effects of illness) and, critically, be scalable and cost-effective. Pupillometry is a tool that satisfies both criteria. Pupil metrics can serve as an objective readout of cognitive and emotional processes affected by schizophrenia^2^^,^^3^ such as attention,^4^^,^^5^ cognitive control,^6^^,^^7^ and emotional regulation.^8^ Furthermore, the neural circuits involved in innervating the muscles that control pupil size are well-delineated, and a rich body of clinical and pre-clinical evidence describes the neuromodulatory basis of pupil dynamics. Relatedly, autonomic nervous system activity, which is dysregulated in schizophrenia,^9^ is reflected in pupil size.^10^ Thus, pupil readouts can provide a window into central illness mechanisms. Finally, pupillometry is non-invasive and can be easily integrated into routine care visits or even using mobile technology.^11^

Current-day pupillometry studies into schizophrenia have largely focused on pupil dilation during cognitively demanding tasks. In neurotypical individuals, dilation increases as task demands increase, until capacity is reached, after which task disengagement occurs together with pupillary constriction. Individuals with schizophrenia show reduced dilation prior to^12–14^ and during^14–18^ cognitively demanding tasks, which is interpreted as reflecting diminished effort. This interpretation aligns with the broader theory that schizophrenia involves deficits in “willed activity,” or the ability to link goals with actions required for their initiation.^19^ Such deficits are emblematic of the cognitive and motivational deficits that characterize the negative syndrome. Indeed, blunted cognitive dilation in people with schizophrenia has been associated (albeit, inconsistently) with negative symptom severity.^12^^,^^16^^,^^17^ Thus, blunted pupillary dilation may form a biomarker of motivational deficits in schizophrenia, rooted in diminished effort allocation.

An older, separate set of findings revealed altered pupillary response to light (ie, pupil light reflex; PLR) in individuals with schizophrenia, including reduced constriction amplitude,^20–22^ longer constriction latency,^21^ and slower re-dilation following light offset.^23^ Intriguingly, we recently found that, like blunted cognitive dilation, reduced constriction to light is related to negative symptoms in individuals with schizophrenia.^24^ To interpret this shared relationship with negative symptoms, we speculated that these pupillary alterations may stem from a common underlying mechanism.^24^ Constriction to light occurs as signals from the retina project to the pretectal olivary nucleus (PON), which innervates the parasympathetic (“rest-and-digest” branch of the autonomic nervous system) Edinger-Westphal (EW) nucleus that projects to the sphincter muscle.^25^ Primate studies indicate the PON also receives top-down cortical inputs from prefrontal and parietal regions^26–29^ that dynamically modulate constriction to light based on attention, prediction, or task demands.^30–32^ Conversely, cognitive dilation is thought to arise from cortically driven (anterior cingulate cortex/dorsolateral prefrontal cortex^33–35^) projections to the locus coeruleus (LC), engaging the sympathetic “fight-or-flight” branch of the autonomic nervous system to initiate dilation. Locus coeruleus output may also inhibit the parasympathetic EW nucleus responsible for PLR constriction and could thereby reduce this reflex.^36^ As a parsimonious mechanistic explanation for blunted PLR and blunted dilation in schizophrenia, therefore, we hypothesize cortical dysregulation may disrupt autonomic control via 2 pathways: (1) chronically inhibiting the PON/EW constriction pathway, reducing parasympathetic tone and blunting PLR; and (2) blunting engagement of the LC during cognitive tasks, thereby impairing sympathetic-driven pupil dilations. If this is the case, blunted PLR constriction and blunted cognitive dilation should be related in the same individuals with schizophrenia.

This theory shaped the aims of the current study. We first aimed to replicate findings of blunted cognitive dilation and blunted light constriction, which are related to negative symptom severity. Critically, we then tested whether blunted dilation and blunted constriction are related in the same individuals. Such a relationship may suggest a shared mechanism underlying these 2 sets of findings, providing a parsimonious explanation for both. Moreover, such an association could enable a more nuanced understanding of the alterations in the neural networks that regulate pupil size changes in schizophrenia and their connection to clinical outcomes and symptoms.

## Methods

### Overview

Pupil dilation in preparation to make an eye movement was measured in participants with schizophrenia (SZ) spectrum disorders and demographically matched healthy controls (HC) while they performed an eye movement task. Pupil light reflex was measured in HC and SZ using a light stimulation protocol. Preparatory dilation and PLR were measured during separate sessions that may or may not have occurred on the same day. The samples of HC and SZ in these 2 studies were largely overlapping (see Tables S1, S2 and S3).

### Participants

Eighty-four individuals with schizophrenia (SZ) spectrum disorders and 69 HC completed a saccadic double-step task in which we measured preparatory pupil dilation, and/or the PLR task. Data were collected as part of a superset of a previously published sample^24^: A total of 9/49 (18.4%) HC and 22/77 (28.6%) SZ were new participants. Participants were recruited from outpatient mental health facilities, existing research registries and subject pools, and community advertisements in the greater Lansing, MI area and were between the ages of 19 and 59. Diagnoses for all groups were based upon the electronic version of the Structured Clinical Interview for Diagnostic and Statistical Manual of Mental Disorders, Fifth Edition (SCID-5) as well as medical records and informant reports when necessary and available. Groups were matched on age, race, ethnicity, and gender (see Table 1). Healthy controls had significantly more years of education *t*(121) = −7.24, *P* < .001 than HC.

**Table 1:** Demographics Information for All Included Participants Who Completed the Saccadic Double-Step and/or the Pupil Light Reflex

	Diagnostic category		Analysis		
	SZ (*N* = 77)	HC (*N* = 48)	Test	*P*	*Post hoc*
Age	35.04 (11.36)	36.00 (10.48)	*t*(123) = −0.47	.64	
Gender (F/M/O)	47/27/3	28/19/1	*X^2^*(3, 125) = 3.67	.29	
Hispanic/Latino (Y/N)	7/70	2/46	*X^2^*(1, 125) = 1.07	.30	
Race			*X^2^*(5125) = 8.81	.12	
Native American	1	0			
Asian	0	4			
Black	23	10			
White	45	30			
Multiracial	4	3			
Other	4	1			
Years of education	13.29 (2.41)	16.64 (2.62)	*t*(121) = −7.24	<.001	SZ < HC
Years of illness	11.06 (9.21)				
IQ	96.66 (23.27)	104.00 (23.82)	*t*(116) = −1.65	.10	
CPZ dosage	366.45 (594.56)				
SANS total	27.30 (19.39)				
SANS MAP	8.72 (7.22)				
SANS EXP	9.35 (8.65)				
BPRS total	42.72 (12.28)				
SAPS total	19.64 (17.57)				

Abbreviations: BPRS: Brief Psychiatric Rating Scale; CPZ: chlorpromazine equivalent dosages; EXP: expressive subscale; HC: healthy controls; MAP: motivation and pleasure; SANS: scale for the assessment of negative symptoms; SAPS: Scale for the Assessment of Positive Symptoms; SZ: schizophrenia.

### Exclusion Criteria

Exclusion criteria for all participants included age < 18 or >60, a history of head injury with loss of consciousness >1-hour, diagnosed neurological disorder, moderate or severe substance use disorder within the past 6 months, estimated premorbid IQ < 70 using the Wechsler Test of Adult Reading,^37^ and vision that was not normal or corrected to normal. Healthy controls were additionally excluded for current history of mental illness or psychotropic use, and first-degree relatives with a history of schizophrenia spectrum or bipolar disorder. All participants gave written informed consent and were reimbursed for participation. The study was approved by the Michigan State University Institutional Review Board.

### Clinical and Cognitive Assessments

Positive and negative symptoms of schizophrenia were assessed using the following measures: The Scale for the Assessment of Positive Symptoms (SAPS),^38^ and the Scale for Assessment of Negative Symptoms (SANS),^39^ respectively. Two subscales were derived based on a previous factor analytic study of the SANS^40^: The expressive subscale (EXP) comprising blunted affect and alogia, and the motivation and pleasure subscale (MAP) comprising anhedonia, asociality, and avolition. General psychiatric symptoms were evaluated with the Brief Psychiatric Rating Scale (BPRS).^41^ To account for potential medication confounds (eg, the effects of medication on pupil size,^42^ chlorpromazine (CPZ) equivalent dosages were calculated for each patient.^43^ Premorbid IQ was assessed using the Wechsler Test of Adult Reading.^37^

### Experimental Paradigms and Procedures

#### Double-Step Task

Cognitive dilation was measured during the pre-stimulus fixation (ie, preparatory) period of a saccadic double-step task. Eye position and pupil diameter were measured using the EyeLink 1000 (SR Research) eye tracker, at a sampling rate of 500 Hz with average gaze position error < 0.5°, noise limited to <0.01° and minimum amplitude criterion (2° visual angle). Pupil diameters (originally in arbitrary units) were converted to millimeters using a calibration curve. We recorded the arbitrary-unit responses for artificial pupils of known diameters (4, 6, 8, 10, and 12 mm) and fitted a polynomial function to those data to map arbitrary units onto true pupil size (in mm).

All participants were tested using an identical setup. Participants were seated 59 cm from a 20-inch CRT monitor (1280 x 960 resolution; 85 Hz refresh rate), with head position stabilized using a chin and forehead rest. The Eyelink camera was mounted at a 24° upward angle and 9.25 inches from the screen, with its position locked for all sessions. As such, distance and focus of recording optics were identical across all subjects and all sessions. All participants were tested in a fully dark room (0.2Lux of ambient light) with no source of light except the computer monitor, which was at minimum brightness (uniform black background: RGB 0,0,0; front-panel brightness set to 0). In addition, a 3-stop neutral density filter was used to reduce stray monitor light. Screen luminance at the participant’s eye position was confirmed using a photometer (Konica Minolta LS-100): ~2.03Lux for the central fixation point and ~0.01Lux for the screen background. MATLAB Psychophysics and EyeLink toolboxes (MathWorks, Portola Valley, CA) were used to present stimuli and collect responses on the double-step task.

**Figure 1:**
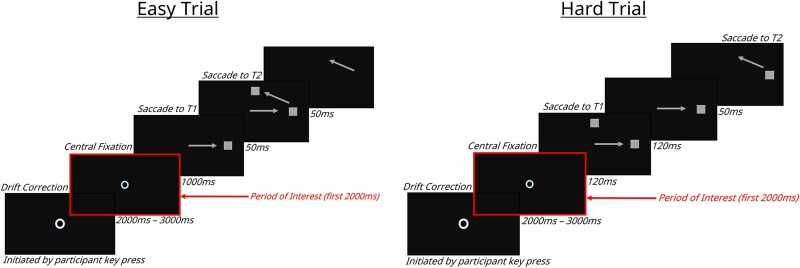
Visual Representation of Easy and Hard Trials on the Saccadic Double-Step. Participants were asked to make a visual saccade to T1 first followed by T2

The double-step task (Figure 1) comprised 2 trial types: easy and hard. For each trial type, 2 visual targets were flashed at a location 12° of visual angle from fixation. Participants were instructed to look at these 2 targets presented in succession, as quickly as possible. On easy trials, the first target (T1) stayed on the screen for 1000 ms. On hard trials, T1 was presented for 120 ms. The second target (T2) always flashed for 50 ms.

Individuals were first asked to initiate a calibration procedure by pressing the space bar. After calibration was completed and prior to starting each trial, a drift check procedure required participants to fixate on a central annulus, representing 2.5% of screen size, and press the space bar. If the eye position exceeded a maximum distance from the annulus, the procedure was repeated. If the procedure failed a second time, the experimenter re-performed the calibration procedure. Following a successful drift check, there was a fixation period of a random duration between 2 and 3 s prior to target presentation. The fixation stimulus was a central white annulus, representing 1.5% of screen size.

Trial types were randomized within blocks. Participants completed 4-8 experimental blocks, with each block consisting of 96 trials. Participants were given no a priori indication as to upcoming trial difficulty. If T1 accuracy was below 60% on a given block, additional blocks were performed until a total of 4 valid blocks were achieved. As double-step task performance was not a measure of interest, all complete blocks across all participants were included in our analyses.

#### Light Stimulation Protocol

Prior to stimulation, participants underwent a standardized 10-s pre-stimulus dark adaptation period in the same dark room (0.2Lux of ambient light) as the double-step task. Following adaptation, the PLR was measured twice for each eye using a handheld NeurOptics PLR-3000 pupillometer (30 Hz sampling rate, accuracy ±0.03 mm). The PLR-3000’s illumination port—a 35 mm circular aperture positioned approximately 22 mm from the corneal apex (∼77° visual angle subtended at the eye)—delivered a 1-s, 451-lux light pulse to evoke constriction, followed by 4 s of darkness to track re-dilation.

Participants were instructed to keep their eyes open, look straight ahead, and blink minimally throughout the 5-s light protocol. We aimed to collect 4 valid measurements per person. There was an average inter-trial interval of 17 s. If the pupillometer flagged the data as unusable, the measurement was repeated. Measurements were averaged across trials to improve the signal-to-noise ratio.

### Data Analysis

#### Double-Step Task

Analysis of pupil dynamics was conducted using MATLAB 2022b. As described above, each trial was initiated by the participant’s key press that formed the end of a successful drift check procedure and that instantly triggered an on-screen change from the drift-check annulus to the task’s initial central fixation spot, which indicated to the participant that they would soon be presented with visual targets to which they should make an eye movement. This fixation window varied randomly between 2 and 3 s on each trial to control for anticipatory effects. The change in pupil size during the first 2 s of the fixation window was our measure of interest. In addition, due to the lack of a priori signaling of upcoming trial difficulty, no differences in preparatory pupil between easy and hard trials were anticipated. As such, trial difficulty was not subsequently considered as a variable of interest.

Prior to any statistical analysis, pupil measurements were smoothed using a low-pass Butterworth filter with a cutoff frequency of 100 Hz and down sampled using a bin size of 20 ms. Pupil dilation was then quantified on each trial as the difference in mean pupil diameter between the last 100 ms of the fixation period and baseline (mean diameter in the first 100 ms after the start of central fixation). As pupil diameter is significantly influenced by both blinks^44^ and gaze position,^45^ trials in which the subjects blinked (full or partial blink) or deviated from fixation by more than 2° of visual angle were excluded from all analyses. Only trials with blinks/artifacts during the dilation period of interest (0-2000 ms relative to fixation onset) were excluded. Full blinks were identified using the automated Eyelink procedure. Partial blinks not captured by the built-in blink detection algorithm were identified by computing velocity traces for all remaining trials across all participants. Trials showing abnormally large positive or negative velocities in subsequent bins (5 standard deviations above or below the mean average velocity between 2 bins for all trials across all participants) were then removed. In addition, trials in which baseline pupil values fell above or below 4 standard deviations from mean baseline pupil values at the subject level were also removed to control for blinks occurring during the transition from the drift correction to the central fixation cross). Consistent with Mathôt et al.,^46^ participants with excessive missing data were excluded to avoid biased estimates; participants with fewer than 10 usable trials were excluded from analyses. As a result, data from 15 SZ and 7 HC were removed from all analyses. Information about excluded participants can be found in Tables S2 and S3.

There were no significant differences in individuals included or excluded from the study for either group (SZ or HC). SZ and HC displayed similar proportion of trials with blinks (*M* = 0.67, SD = 0.25; *M* = 0.63, SD = 0.26, respectively), *t*(104) = 0.86, *P* = .39. HC did, however, display significantly greater proportion of deviations from fixation (>2° of visual angle) than SZ (*M* = 0.61, SD = 0.13; *M* = 0.48, SD = 0.14, respectively), *t*(104) = −4.48, *P* < .001.

Following data cleaning, we retained an average of 68.8 (SD = 49.87) and 85.95 usable trials (SD = 59.79) for HC and SZ, respectively. An independent samples *t*-test revealed no significant difference in the number of usable trials between diagnostic groups, *t*(89) = –1.43, *P* = .08.

#### Light Stimulation Protocol

Analyses of pupil dynamics were conducted using MATLAB 2022b. In line with Fattal et al.,^24^ baseline pupil size was calculated as the mean diameter during the first 150 ms of the measurement, which is prior to the onset of the pupil response to even an intense light stimulus.^47^ Constriction amplitude was quantified as the absolute value of the difference between the maximally constricted pupil diameter and initial diameter. Pupil measurements were smoothed using a low-pass Butterworth filter with a cutoff frequency of 4 Hz. Velocity and acceleration traces were then calculated to identify blinks (successive >3x interquartile range negative-then-positive outliers in the velocity trace occurring within 0.5 s of one another). If blinks occurred before initial constriction to light, the entire trial was removed. If blinks occurred after redilation onset, dilation metrics were removed but constriction metrics were kept. Then, pupil size time courses were averaged across each measurement trial for each subject. Information about excluded participants can be found in Tables S2 and S3. There were no significant differences in individuals included or excluded from the study for either group (SZ or HC).

### Statistical Analysis

Prior to our main analyses, independent samples *t*-tests and Chi-square tests of independence were conducted to compare demographic and clinical characteristics of the SZ group to the HC group. All analyses were conducted using SPSS Statistics Version 28.0 (IBM, Armonk, NY) and missing data were handled using pairwise deletion. All model assumptions were met.

#### Group Differences in Preparatory Dilation and Constriction Amplitude

Independent samples *t*-tests were conducted to investigate differences between SZ and HC in mean preparatory pupil dilation (averaged across trials for each subject) on the double-step task and constriction amplitude on the PLR.

#### Symptom Correlates

For the SZ group, Pearson’s correlations were used to assess the association between (1) constriction amplitude on the light stimulation protocol and symptom measures and (2) mean preparatory dilation (averaged across trials for each subject) during the double-step task and symptom measures.

#### Relating Preparatory Dilation and Pupil Light Reflex in HC and SZ

Correlations between constriction amplitude on the light stimulation protocol and mean preparatory pupil dilation on the double-step task were evaluated using Pearson’s correlations for SZ and HC separately. Unduly influential points were identified via Cook’s distance >1, and studentized residuals > |2|; these were excluded to ensure robustness of the correlations. Once complete, partial correlations were conducted for our SZ group using CPZ dosages to account for the effects of antipsychotic medication on pupil size.

To determine whether the correlation between constriction amplitude and preparatory dilation differed significantly between the SZ and HC Groups, Fisher’s *R*-to-*Z* transformation was performed. This analysis was repeated after controlling for CPZ dosage to account for the effects of antipsychotic medication on pupil size.

## Results

### Group Differences in Preparatory Dilation and Constriction Amplitude

**Figure 2:**
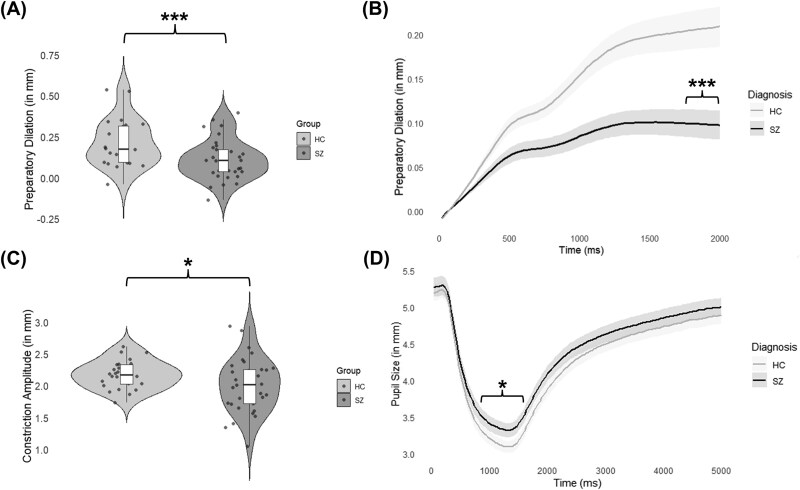
^***^Indicates a Significant Difference Between Groups at *P* < .001; ^*^Indicates a Significant Difference Between Groups at *P* < .05. (A) Violin plot depicting differences in preparatory dilation (in mm) between healthy controls (HC) and individuals with schizophrenia (SZ) spectrum disorders. SZ show blunted preparatory dilation relative to HC. (B) Pupil dilation during the fixation period of the double-step task for SZ and HC. Shaded error bars represent standard errors. SZ showed significantly blunted pupil dilation during the last 100 ms of measurement when compared to HC. (C) Violin plot depicting differences in constriction amplitude (in mm) between HC and SZ. SZ show blunted constriction amplitude relative to HC. (D) Pupil diameter during light stimulation for SZ and HC. Shaded error bars represent standard errors. SZ showed significantly blunted constriction amplitude when compared to HC

Individuals with schizophrenia (SZ) spectrum disorders showed significantly blunted preparatory dilation (*t*(89) = 4.06, *P* < .001, 95% CI [0.06, 0.16] representing mean differences between HC and SZ in preparatory dilation, *d* = .71; Figure 2A) and constriction amplitude (*t*(90) = 2.21, *P* = .03, 95% CI [0.02, 0.36] representing mean differences between HC and SZ in constriction amplitude, *d* = .09; Figure 2C) when compared to HC. A visual representation of pupil dilation during the double-step task (Figure 2B) and constriction amplitude during the PLR (Figure 2D) are provided below. See Table 2 for full PLR results.

**Table 2:** Comparison of NeurOptics PLR 3000 Pupillary Metrics in SZ and HC

	Diagnostic category		Data analysis		
	SZ mean (SD)	HC mean (SD)	Test	*P*	Effect size (*d*)
Initial diameter (mm)	5.23 (1.04)	5.29 (0.66)	*t*(90) = 0.36	.72	0.01
End diameter (mm)	3.24 (0.75)	3.11 (0.53)	*t*(90) = −0.89	.38	−0.04
Constriction amplitude (mm)	1.99 (0.52)	2.18 (0.31)	*t*(90) = 2.21	.03	0.09
Dilation latency (s)	0.25 (0.14)	0.29 (0.12)	*t*(89) = 1.68	.09	0.18
Dilation velocity (mm/s)	0.46 (0.12)	0.51 (0.09)	*t*(89) = 1.94	.06	0.10
Max dilation velocity (mm/s)	1.81 (0.39)	1.91 (0.53)	*t*(89) = 0.99	.32	0.05
Time to 50% recovery (s)	2.29 (0.40)	2.38 (0.39)	*t*(89) = 1.09	.28	0.04

Abbreviations: HC: healthy controls; PLR: pupil light reflex; SZ: schizophrenia.

### Symptom Correlates

Replicating prior findings in a subsample of the current SZ sample, constriction amplitude was negatively associated with SANS Total (*r*(42) = −0.37, *P* = .02, 95% CI [−0.61, −0.09]) and SANS motivation and pleasure (MAP; *r*(42) = −0.43, *P* < .01, 95% CI [−0.63, −0.18]) but not SAPS Total (*r*(42) = −0.18, *P* = .25, 95% CI [−0.42, 0.07]), SANS Expressive (*r*(42) = −0.20, *P* = .19, 95% CI [−0.51, 0.11]), nor BPRS Total (*r*(42) = −0.28, *P* = .07, 95% CI [−0.54, 0.01]).

Preparatory dilation was not associated with SAPS Total (*r*(44) = −0.12, *P* = .43, 95% CI [−0.33, 0.09]), SANS Total (*r*(44) = 0.04, *P* = .79, 95% CI [−0.27, 0.37]), BPRS Total (*r*(44) = −0.11, *P* = .48, 95% CI [−0.37, 0.17]), SANS MAP (*r*(44) = −0.01, *P* = .98, 95% CI [−0.31, 0.36]), nor SANS EXP (*r*(44) = 0.06, *P* = .68, 95% CI [−0.27, 0.41]).

### Relating Pupil Dilation and Constriction Amplitude in HC and SZ

**Figure 3:**
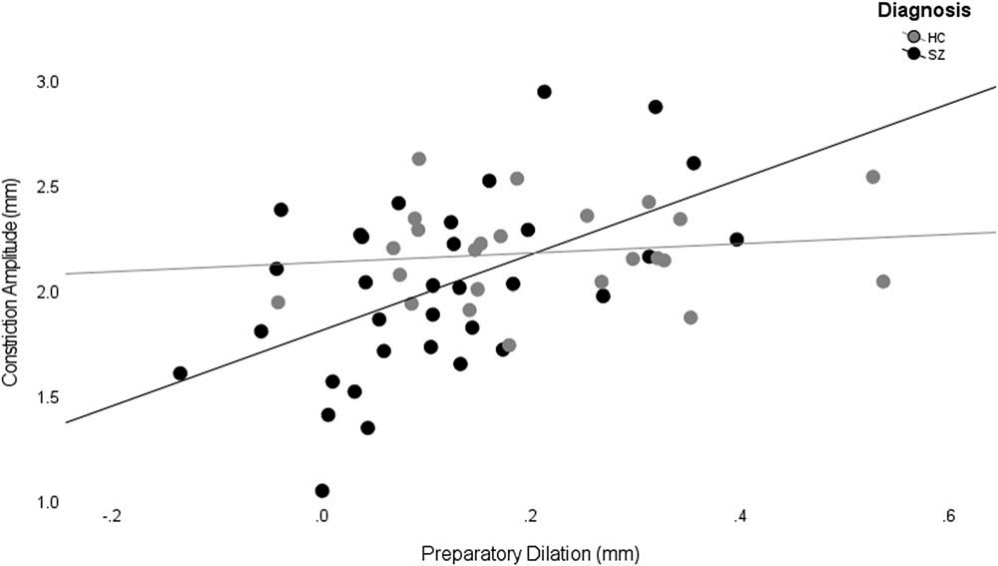
Correlation Between Preparatory Dilation (in mm) and Constriction Amplitude (in mm) for Schizophrenia (SZ) Spectrum Disorders and Healthy Controls (HC) Separately. SZ showed a significant positive correlation between amplitude and preparatory dilation that was not present in HC

Preparatory pupil dilation was positively associated with mean constriction amplitude in SZ (*r*(33) = 0.52, *P* < .01, 95% CI [0.28, 0.71]) but not in HC (*r*(24) = 0.14, *P* = .51, 95% CI [−0.28, 0.53]; Figure 3), indicating that individuals with schizophrenia who showed greater constriction to light also showed greater preparatory pupil dilation. No univariate outliers were detected for either variable across both groups, and all Cook’s distances in a regression of preparatory pupil dilation predicting constriction amplitude were <1, indicating no highly influential cases. Three observations (2 SZ, 1 HC) exhibited studentized residuals >|2|; excluding these did not change the correlation (*r*(31) = 0.49, *P* < .01, 95% CI [0.17, 0.72]). Thus, our reported correlation is robust to outlier-screening procedures.

Using partial correlation, this relationship in SZ persisted when controlling for normalized antipsychotic dose (*r*(30) = 0.53, *P* < .01, 95% CI [0.30, 0.77]). Fisher’s *R* to Z indicated that the relationship between constriction to light and preparatory dilation was not significantly different between HC and SZ both with (*z* = 1.54, *P =* .06) and without (*z* = 1.53, *P =* .06) controlling for CPZ dosage.

### Additional Control Analysis

After completing the analyses described above, we identified a potential confound that might trivially explain the correlation of Figure 3. In the double-step paradigm from which we isolated measures of preparatory dilation, the drift-check and fixation annuli differed in size (2.5% and 1.5% of the screen, respectively), meaning that the onset of the preparatory period in our double-step task was marked by a reduction in overall screen luminance. This means that the observed dilation, which we have referred to as preparatory dilation, may also include a pupil dark response (related to the luminance change) along with the cognitive (ie, preparatory) pupil dilation. One potential explanation of the correlation of Figure 3, then, is that participants with a more pronounced PLR (*y*-axis), also have a more pronounced contribution of a pupil dark response to our dilation measure (*x*-axis). We conducted control analyses with *n* = 5 additional HC participants to assess this possibility. Specifically, participants completed 2 runs of the double-step task that differed only in fixation annulus size: One with the original size (luminance change condition) and another in which fixation and drift correct annuli were equivalent in size (no-luminance change condition; both 2.5% of screen size). Condition order was counterbalanced across participants.

**Figure 4:**
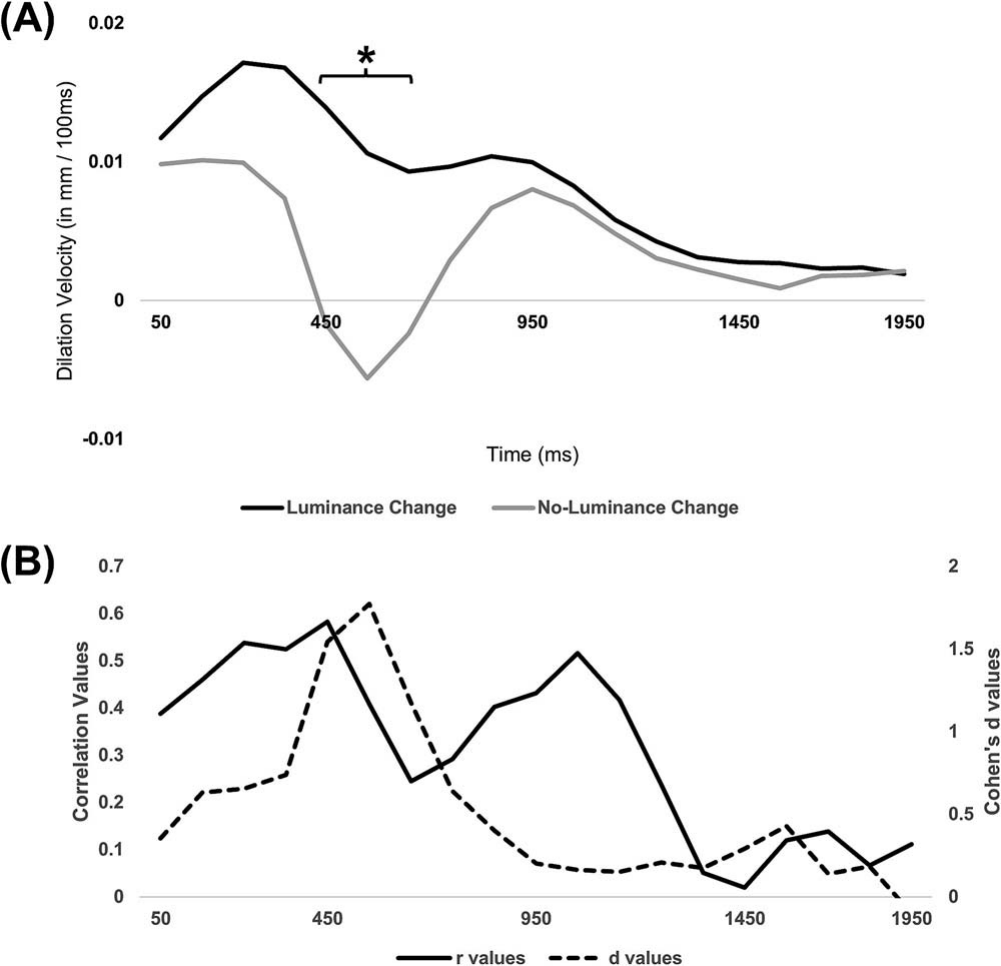
(A) Preparatory Dilation Velocity (in mm / 100 ms) as a Function of Time (ms) on the Double-Step Task for Luminance Change (Original Annuli) and No-Luminance Change (Size-Equivalent Annuli) Control Conditions. Preparatory dilation velocity is significantly greater in luminance change than no-luminance change from 400 to 600 ms. (B) The solid line tracks changes in the correlation strength (in *r* values) over time (in ms) between preparatory dilation and constriction amplitude within the main experiment’s SZ group. The dashed line tracks changes in the effect size (*d* values) over time (in ms) for paired samples *t*-tests comparing the no-luminance change and luminance change control conditions. Notably, the time course of the correlation strength does not match that of the effect size values

Pupil size time courses (in mm) were preprocessed as described above and averaged across trials and across participants for each fixation size control condition (ie, luminance change vs no-luminance change). The velocity of the time courses was then calculated as a function of time to investigate the contributions of light-driven versus preparation-related pupil size changes. We computed these rates of pupil size change in 100 ms segments. The result is shown in Figure 4A.

Using paired samples *t*-tests, we then searched for distinct 100 ms segments when preparatory pupil dilation velocity significantly differed between the 2 fixation size control conditions. Both fixation size control conditions involved the same task, yet the 2 differed in stimulus luminance characteristics. Thus, time periods in which pupil size change differs between the 2 fixation size control conditions are likely periods in which a pupil dark response substantially contributes to pupil dilation in the luminance change condition. We confirmed that such time periods were present, with the largest (and only significant) differences between the 2 conditions arising around 400-600 ms after the fixation annulus change (*t*(4) = 3.45, *P* = .03, *d* = 1.54 for the 400-500 ms window; *t*(4) = 3.96, *P* = .02, *d* = 1.77 for the 500-600 ms window; *t*(4) = 2.62, *P* = .06, *d* = 1.17 for the 600-700 ms window). During those time windows, the pupil dilated more rapidly for the luminance change condition than the no-luminance change condition, presumably because of the contribution of a pupil dark response in the former condition. This is consistent with other work showing that the rate of pupil dark dilation peaks around that time.^48^^,^^49^

The question we wished to answer is whether the correlation shown in Figure 3 is driven by a contribution of this pupil dark response to the dilation that we have termed preparatory dilation, and that was measured using the same fixation annulus as used in the luminance change control condition. To answer this question, we re-assessed the correlation of Figure 3, but now for individual time segments of the preparatory dilation curves measured in our main experiment. Specifically, for each time window following fixation annulus change, we computed the correlation between pupil dilation rate, and the same pupil light constriction amplitude measure as used in Figure 3. To test if periods with larger correlation strengths coincided temporally with periods with more dark response expression, we compared these *r* values to effect sizes (Cohen’s *d* values) derived from the paired-samples *t*-tests comparing pupil velocity between the no-luminance change and luminance change control conditions. Both the *r* values and the Cohen’s *d* values are shown on the same time axis in Figure 4B.

If the correlation of Figure 3 were driven by a contribution of the pupil dark response to the preparatory dilation curves, then we should find the strongest correlations around the 400-600 ms time points, when Figure 4A and B shows the largest pupil dark response rate and effect size. Moreover, the correlations should be absent after about 1100 ms, when Figure 4A and B shows barely any evidence for a pupil dark response at all (ie, no difference between the luminance change and no-luminance change control conditions).

The results shown in Figure 4B do not match these predictions. The correlation between dilation rate and constriction amplitude actually drops, rather than peaks, around 500-600 ms; precisely when the difference in pupil velocity between control conditions is maximal (*d* = 1.77). Also, the correlation is positive for some time after 1100 ms, even while the effect size difference between control conditions is near its lowest. Collectively, these results suggest that changes in luminance induced by the size of the annulus in our main experiment are not the primary driver of the observed correlation between preparatory pupil dilation and constriction amplitude in the SZ sample (Figure 3). Instead, they support a mechanistic link rooted in cognitive/arousal processes.

## Discussion

The current study yielded 2 major findings. First, we replicated prior findings in individuals with schizophrenia of: (1) blunted constriction to light that is related to negative symptoms in a superset of a previously published^24^ sample, and, (2) blunted dilation in preparation to act in an independent sample. Critically, we extended prior work and found that blunted constriction was correlated with blunted cognitive dilation in individuals diagnosed with schizophrenia. This correlation may suggest a common mechanism underlying these 2 sets of findings, thereby inviting a reinterpretation of altered cognitive dilation and a parsimonious explanation for these pupil abnormalities in schizophrenia. However, we cannot rule out unmeasured third variables that drive this correlation. In the following section, we interpret our findings in the context of prior work in individuals with schizophrenia and physiology of pupil size dynamics. We also address limitations and future directions.

Our finding of blunted pupillary constriction in response to light is consistent with our prior findings in a subset of the current sample^24^ and with a large body of prior work.^20^^,^^22^^,^^50^^,^^51^ More importantly, we replicated significant associations between blunted constriction and increased negative symptom severity—particularly motivational negative symptom severity. We also replicated findings of reduced preparatory dilation during a simple oculomotor task. This finding replicates prior reports of blunted pupillary dilation while participants are engaging in a cognitive task^15–18^^,^^52^ and preparing to execute a cognitively effortful response (eg, inhibiting a pre-potent response).^12–14^ Because the current task simply required visually guided saccades, our findings extend prior work to show that blunted dilation is even observed during the preparation of a simple motor action in people with schizophrenia.

Importantly, however, we did not replicate previous findings that blunted preparatory dilation was related to more severe negative symptoms. Motivational negative symptoms, which are characterized by reduced engagement in goal-directed behavior, have previously been associated with blunted cognitive-related dilation.^16–18^^,^^52^^,^^53^ Blunted cognitive-related is thought to serve as a proxy measure of diminished effort in schizophrenia.^15–18^^,^^52^ As such, deficits in effort may underlie motivational negative symptoms of schizophrenia. Difficulty in assessing the subjective costs of cognitive effort,^54^ anticipating future rewards,^55^ and increases in defeatist performance beliefs (ie, over-generalized negative thoughts about one’s ability to successfully perform goal-directed behavior^56^^,^^57^) have been proposed as explanations for this association. There are several factors that may explain why we were unable to replicate dilation-negative-symptom relationships in the current study. Negative symptom presentations are often variable, fluctuating over time,^58^ illness stage,^59^ and in response to medication.^60^ This variability would limit their usefulness as reliable indicators of underlying cognitive and physiological processes that confer *vulnerability* to symptoms, which may be captured by pupillary response. Thus, the inconsistent associations observed in our study may partly reflect the dynamic and changing nature of symptoms themselves, rather than the value of pupil metrics as tools for identifying mechanisms of schizophrenia. Alternately, such relationships may only emerge when the pre-task dilation reflects the preparation to engage in a cognitively demanding task; this hypothesis could be tested by examining dilation-symptom relationships as a function of expected difficulty of upcoming cognitive demands.

The most important finding of the current study was the correlation between preparatory dilation and pupil light constriction amplitude, which was only significant in individuals with schizophrenia. Individuals with schizophrenia showing less dilation in preparation to act also showed less constriction to light. This is the first study, to our knowledge, to show a significant association between these 2 sets of previously independently established findings. While blunted preparatory dilation has typically been interpreted as reflecting reduced information processing or effort allocation in individuals with schizophrenia, blunted pupil constriction has typically been interpreted through the lens of autonomic dysregulation. Light indirectly innervates the parasympathetic EW nucleus, which projects onto the sphincter muscles that constrict the pupil. However, pupil constriction is likely not purely reflexive. In primates, reactions to light are dynamically regulated by prefrontal and parietal cortices^26–29^ which send top-down signals to the EW nucleus to initiate constriction based upon attention or predictions^30–32^ and not strictly sensory input (eg, light). This modulation is evident in several scenarios: For instance, the pupil constricts when attention is directed toward light versus dark stimuli, even as overall luminance remains unchanged^32^; it also changes when individuals mentally visualize shapes that differ in brightness or complexity,^61^ despite the absence of external visual input; and it constricts when visualizing pictures of highly luminous objects (eg, the sun), despite no actual increase in luminance.^62^ Thus, blunted pupillary constriction to light in people with schizophrenia may reflect disrupted cortical modulation of autonomic reflexes. However, this interpretation requires direct validation (eg, neuroimaging of cortical-EW interactions).

While altered top-down control of the EW nucleus may explain blunted pupil constriction to light, it does not fully account for blunted preparatory dilation, nor for the association between blunted PLR and blunted task-related dilation. Task-related pupil dilation, operationalized as a marker of cognitive effort,^63^ is thought to be regulated via activation of the sympathetic nervous system.^64^ In rodents, sympathetic dilation is driven by the LC-norepinephrine (LC-NE) system, which is itself activated by higher cortical regions such as the anterior cingulate cortex and the dorsolateral prefrontal cortex.^33–35^ Critically, some studies also suggest that the LC-NE system may suppress the EW nucleus responsible for pupil constriction.^65^

However, these theories do not rule out the possibility that direct exertion by the prefrontal and parietal cortex on the EW nucleus in primates—such as in the case of cognitively driven pupil constriction described above—is the *primary* mechanism underlying impaired cognitive preparatory dilation in schizophrenia, independent of mediation by the LC-NE system. In schizophrenia, where parasympathetic dysfunction—rather than sympathetic impairment—predominates,^66^^,^^67^ blunted pupil responses may reflect chronic dysregulation of direct cortical input to the EW nucleus. Sustained processing and performance during difficult cognitive tasks requires inhibition of the parasympathetic pathway,^68^ and failure to suppress this system is related to worse performance on tasks of central executive function.^69^ Consequently, reduced preparatory pupil dilation may represent impaired cortical disinhibition of the EW nucleus during task demands, rather than deficits in sympathetic engagement. Mechanistically, impaired cortical control over the EW nucleus could simultaneously (a) blunt preparatory dilation by failing to suppress parasympathetic constriction of the iris and (b) attenuate light-elicited constriction by failing to appropriately activate parasympathetic constriction. Together, these observations suggest a unified explanation: Cortically driven parasympathetic dysregulation alone—rather than independent sympathetic and parasympathetic deficits—may drive the observed correlation between blunted task-related preparatory dilation and light-related pupillary constriction.

Regardless of the precise mechanism underlying this observed correlation, it is clear that cognitive task-related pupil dilations are unlikely to reflect purely effort-related processes. Instead, such dilations likely also (or instead) reflect processes that integrate more reflexive autonomic components—particularly parasympathetic withdrawal—with higher-order cognitive functions. Recognizing this interplay is critical for evaluating the validity of reverse inferences commonly drawn from pupillometry data; specifically, interpreting pupil dilation as a direct index of cognitive effort requires a clear understanding of the underlying physiological mechanisms, particularly when such responses are altered or blunted.

However, other reasons for this association between pupillary constriction in response to light and preparatory dilation must be considered. For example, the pupil response during the preparatory period may reflect, in part, redilation as a result of a change in luminance upon fixation cue onset. However, control analyses suggest that this cannot explain the observed correlation between preparatory dilation and constriction in response to light. The shared variance in dilation and constriction may also be accounted for by a third variable, eg, antipsychotic medication use, which can cause mydriasis (pupil dilation) and miosis (pupil constriction).^42^ This may also explain the lack of a significant correlation in HC. However, analyses including CPZ dosage replicated all findings, arguing against medications being a primary explanation for dilation-constriction relationships in people with schizophrenia. Nevertheless, there may be other unmeasured variables that may drive this association.

The current study has several limitations that should be acknowledged. First, the participants included in the correlation between task-related dilation and constriction in response to light was a subset of the total sample who had usable data for both tasks. The smaller final sample likely limited our statistical power to detect subtle effects, though sensitivity analyses confirmed that results were not driven by outliers. Second, while control analyses addressed potential confounding by light-initiated pupil dilation, residual effects of luminance changes on the observed outcomes cannot be entirely ruled out. Finally, similar to luminance effects, we cannot fully exclude residual confounding by antipsychotic medication, despite accounting for medication doses in our analyses.

Future studies may expand on the results of the current study in 2 meaningful ways. First, current findings may be replicated at the within-person level to control for person-level factors that may have confounded results. Second, researchers may directly test the hypothesized cortico-EW-nucleus dysregulation in schizophrenia using effective connectivity analyses of fMRI data.

## Conclusion

In conclusion, our findings establish a significant association between blunted constriction to light and blunted cognitively related preparatory dilation in individuals with schizophrenia for the first time, which may hint at a common mechanism. We interpret these findings as potentially reflecting deficits in top-down control of autonomic functions. This preliminary theory merits testing in future studies. In addition, our findings enhance the utility of pupillometry as a clinical biomarker of schizophrenia mechanisms. Implementing pupillometry in future research as well as clinical settings stands to further unveil pathophysiological illness mechanisms and treatment targets for schizophrenia.

## Supplementary Material

SUPPLEMENTAL_TABLES_sgaf017
