# Supplementary material for: Associations Between Blunted Pupillary Constriction and Dilation in Individuals with Schizophrenia: Evidence of a Common Mechanism?
Source: Schizophr Bull Open. 2025 Aug 20;6(1):sgaf017. doi: 10.1093/schizbullopen/sgaf017 (PMC12477605; doi:10.1093/schizbullopen/sgaf017)
Supplement: SUPPLEMENTAL_TABLES_sgaf017 [file supplemental_tables_sgaf017.docx]

SUPPLEMENTAL TABLES

Table 1: Demographics information for all included participants who completed both the saccadic double-step task and the pupil light reflex.

|  | Diagnostic Category | | Analysis | | |
| --- | --- | --- | --- | --- | --- |
|  | SZ (N = 32) | HC (N = 24) | Test | *p* | *Post Hoc* |
| Age | 36.41 (11.12) | 37.40 (9.50) | *t*(54) = -.35 | .73 |  |
| Gender (F/M/NB/O) | 19/11/0/2 | 15/9/0/0 | *X^2^*(2,56) = 1.56 | .46 |  |
| Hispanic/Latino (Y/N) | 3/29 | 1/23 | *X^2^*(1,56) = .56 | .45 |  |
| Race |  |  | *X^2^*(5,56) = 8.83 | .12 |  |
| Native American | 1 | 0 |  |  |  |
| Asian | 0 | 2 |  |  |  |
| Black | 10 | 3 |  |  |  |
| White | 19 | 18 |  |  |  |
| Multiracial | 2 | 0 |  |  |  |
| Other | 0 | 1 |  |  |  |
| Years of education | 13.44 (2.26) | 16.75 (2.66) | *t*(54) = -5.04 | <.001 | HC > SZ |
| Years of Illness | 10.97 (9.06) |  |  |  |  |
| IQ | 97.78 (21.17) | 104.42 (22.94) | *t*(54) = -1.12 | .27 |  |
| CPZ Dosage | 272.18 (301.64) |  |  |  |  |
| SANS Total | 29.77 (20.51) |  |  |  |  |
| SANS MAP | 10.59 (8.13) |  |  |  |  |
| SANS EXP | 9.13 (9.02) |  |  |  |  |
| BPRS Total | 43.75 (13.66) |  |  |  |  |
| SAPS Total | 18.92 (15.47) |  |  |  |  |

Chlorpromazine Equivalent (CPZ) Dosages; Scale for the Assessment of Negative Symptoms (SANS); Brief Psychiatric Rating Scale (BPRS); Scale for the Assessment of Positive Symptoms (SAPS).

Table 2: Demographics information for included/excluded participants who completed the saccadic double-step task.

|  | Performance Exclusion Criteria | | Analysis | | |
| --- | --- | --- | --- | --- | --- |
|  | Included | Excluded | Test | *p* | Effect Size (*d* or *V*) |
| Sample Size |  |  |  |  |  |
| SZ | 55 | 15 |  |  |  |
| HC | 36 | 7 |  |  |  |
| Age |  |  |  |  |  |
| SZ | 34.18 (11.06) | 38.73 (11.32) | *t*(68) = -1.41 | .16 | -.13 |
| HC | 37.06 (9.92) | 29.29 (5.74) | *t*(41) = 1.99 | .053 | .22 |
| Gender (F/M/NB/O) |  |  |  |  |  |
| SZ | 34/19/2/0 | 7/8/0/0 | *X^2^*(2, 70) = 2.09 | .35 | .17 |
| HC | 20/16/0/0 | 5/2/0/0 | *X^2^*(1, 43) = 0.61 | .44 | .12 |
| Hispanic/Latino (Y/N) |  |  |  |  |  |
| SZ | 5/50 | 1/14 | *X^2^*(1, 70) = 0.09 | .77 | .04 |
| HC | 1/35 | 0/7 | *X^2^*(1, 43) = 0.19 | .66 | .07 |
| Race |  |  |  |  |  |
| SZ |  |  | *X^2^*(4, 70) = 5.74 | .22 | .29 |
| HC |  |  | *X^2^*(5, 43) = 10.87 | .054 | .50 |
| Native American |  |  |  |  |  |
| SZ | 1 | 0 |  |  |  |
| HC | 0 | 0 |  |  |  |
| Asian |  |  |  |  |  |
| SZ | 0 | 0 |  |  |  |
| HC | 2 | 3 |  |  |  |
| Black |  |  |  |  |  |
| SZ | 19 | 4 |  |  |  |
| HC | 5 | 0 |  |  |  |
| White |  |  |  |  |  |
| SZ | 30 | 8 |  |  |  |
| HC | 26 | 3 |  |  |  |
| Multiracial |  |  |  |  |  |
| SZ | 2 | 3 |  |  |  |
| HC | 2 | 0 |  |  |  |
| Other |  |  |  |  |  |
| SZ | 3 | 0 |  |  |  |
| HC | 1 | 1 |  |  |  |
| Years of education |  |  |  |  |  |
| SZ | 13.36 (2.45) | 13.87 (1.92) | *t*(68) = -.73 | .47 | -.04 |
| HC | 17.00 (2.54) | 17.29 (2.21) | *t*(41) = -.28 | .78 | -.02 |
| Years of Illness | 9.79 (8.79) | 13.64 (11.29) | *t*(65) = -1.37 | .18 | -.36 |
| IQ |  |  |  |  |  |
| SZ | 95.74 (26.07) | 102.21 (9.38) | *t*(66) = -.91 | .37 | -.07 |
| HC | 103.97 (26.18) | 111.14 (6.91) | *t*(41) = -.71 | .48 | -.07 |
| CPZ Dosage | 330.20 (407.54) | 379.41 (410.73) | *t*(67) = -.40 | .69 | -.14 |
| SANS Total | 26.53 (20.75) | 30.40 (19.58) | *t*(68) = -.65 | .52 | -.14 |
| SANS MAP | 10.55 (7.65) | 8.75 (8.68) | *t*(68) = -.91 | .37 | -.23 |
| SANS EXP | 9.16 (8.72) | 8.87 (8.32) | *t*(68) = .12 | .91 | .03 |
| BPRS Total | 43.16 (13.29) | 47.27 (12.76) | *t*(68) = -1.07 | .29 | - .09 |
| SAPS Total | 19.78 (16.13) | 24.27 (19.72) | *t*(66) = -.91 | .37 | -.22 |

Chlorpromazine Equivalent (CPZ) Dosages; Scale for the Assessment of Negative Symptoms (SANS); Brief Psychiatric Rating Scale (BPRS); Scale for the Assessment of Positive Symptoms (SAPS).

Table 3: Demographics information for included/excluded participants who completed the pupil light reflex.

|  | Performance Exclusion Criteria | | Analysis | | |
| --- | --- | --- | --- | --- | --- |
|  | Included | Excluded | Test | *p* | Effect Size (*d* or *V*) |
| Sample Size |  |  |  |  |  |
| SZ | 55 | 11 |  |  |  |
| HC | 36 | 19 |  |  |  |
| Age |  |  |  |  |  |
| SZ | 36.62 (11.34) | 33.18 (10.46) | *t*(64) = .93 | .36 | .09 |
| HC | 37.06 (9.92) | 36.11 (10.72) | *t*(53) = .03 | .97 | -.02 |
| Gender (F/M/NB/O) |  |  |  |  |  |
| SZ | 33/17/3/2 | 6/4/0/1 | *X^2^*(3, 66) = 1.33 | .72 | .14 |
| HC | 23/10/2/1 | 10/1/8/0 | *X^2^*(3, 55) = 13.08 | <.01 | .49 |
| Hispanic/Latino (Y/N) |  |  |  |  |  |
| SZ | 5/50 | 1/10 | *X^2^*(1, 66) = 1.00 | .99 | .00 |
| HC | 2/34 | 3/16 | *X^2^*(1, 55) = 1.58 | .21 | .21 |
| Race |  |  |  |  |  |
| SZ |  |  | *X^2^*(4, 66) = 1.92 | .75 | .17 |
| HC |  |  | *X^2^*(6, 55) = 15.14 | .02 | .53 |
| Native American |  |  |  |  |  |
| SZ | 1 | 0 |  |  |  |
| HC | 0 | 4 |  |  |  |
| Asian |  |  |  |  |  |
| SZ | 0 | 0 |  |  |  |
| HC | 3 | 0 |  |  |  |
| Black |  |  |  |  |  |
| SZ | 15 | 3 |  |  |  |
| HC | 8 | 1 |  |  |  |
| White |  |  |  |  |  |
| SZ | 34 | 6 |  |  |  |
| HC | 22 | 10 |  |  |  |
| Multiracial |  |  |  |  |  |
| SZ | 4 | 1 |  |  |  |
| HC | 1 | 3 |  |  |  |
| Other |  |  |  |  |  |
| SZ | 1 | 1 |  |  |  |
| HC | 1 | 1 |  |  |  |
| Years of education |  |  |  |  |  |
| SZ | 13.25 (2.28) | 13.09 (2.95) | *t*(64) = .21 | .84 | .01 |
| HC |  |  |  |  |  |
| Years of Illness | 12.22 (9.33) | 8.45 (8.87) | *t*(60) = 1.22 | .23 | .32 |
| IQ |  |  |  |  |  |
| SZ | 98.10 (18.18) | 92.27 (33.06) | *t*(61) = .82 | .41 | .06 |
| HC | 103.97 (26.18) | 104.33 (20.23) | *t*(50) = - 1.16 | .25 | -.05 |
| CPZ Dosage | 375.42 (657.16) | 425.87 (435.81) | *t*(63) = -.24 | .81 | -.13 |
| SANS Total | 30.33 (19.82) | 33.09 (24.48) | *t*(63) = -.40 | .69 | -.09 |
| SANS MAP | 10.37 (7.43) | 11.45 (8.98) | *t*(63) = -.43 | .67 | -.10 |
| SANS EXP | 9.70 (9.12) | 11.91 (10.02) | *t*(63) = -.72 | .47 | -.22 |
| BPRS Total | 43.48 (12.79) | 43.10 (13.99) | *t*(62) = .09 | .93 | .01 |
| SAPS Total | 19.56 (18.05) | 25.82 (20.65) | *t*(64) = -1.03 | .31 | -.30 |

Chlorpromazine Equivalent (CPZ) Dosages; Scale for the Assessment of Negative Symptoms (SANS); Brief Psychiatric Rating Scale (BPRS); Scale for the Assessment of Positive Symptoms (SAPS).
